# Transcranial Direct Current Stimulation: Challenges, Opportunities, and Impact on Psychiatry and Neurorehabilitation

**DOI:** 10.3389/fpsyt.2013.00019

**Published:** 2013-03-27

**Authors:** Andre R. Brunoni, Paulo Sergio Boggio, Roberta Ferrucci, Alberto Priori, Felipe Fregni

**Affiliations:** ^1^Clinical Research Center, University Hospital, University of Sao PauloSao Paulo, Brazil; ^2^Cognitive Neuroscience Laboratory and Developmental Disorders Program, Center for Health and Biological Sciences, Mackenzie Presbyterian UniversitySao Paulo, Brazil; ^3^Clinical Center for Neurotechnology, Neurostimulation and Movement Disorders, Fondazione IRCCS “Ca’ Granda” Ospedale Maggiore di MilanoMilano, Italy; ^4^Department of Medical-Surgical Pathophysiology and Transplants Section of Neurosciences, University of MilanMilano, Italy; ^5^Laboratory of Neuromodulation, Spaulding Rehabilitation Hospital, Harvard Medical SchoolBoston, MA, USA

The simplicity of the technique of transcranial direct current stimulation (tDCS) can be observed as it consists of a current generator and two electrodes that are placed over the scalp and can deliver weak direct currents. Despite its simplicity, the field of non-invasive brain stimulation has had a rapid and exponential increase in the past 10 years. It is in fact an “old, new” technique – as external brain electric stimulation with electric currents has been recurrently described in medical literature since ancient times (Brunoni et al., 2011b), although the technique was reappraised only recently after the seminal studies of Priori et al. (1998) and Nitsche and Paulus (2000), which showed that it could modify cortical excitability in a polarity-dependent manner, i.e., while anode induces neuronal depolarization and thus activation of neural networks beneath the electrode, the cathode induces the opposite effects (i.e., hyperpolarization and consequent inhibition). From 1998 onward, several studies showed that tDCS modulates a plethora of behavioral, sensorial, or motor effects according to parameters of stimulation and subjects’ characteristics. Two important characteristics of tDCS – the duration of its effects and its safety – have attracted the attention of a large number of scientists and clinicians. Indeed tDCS effects can last for several hours beyond the period of stimulation in some cases (Fregni and Pascual-Leone, 2007) and induce changes in brain biochemistry (Rango et al., 2008). In addition, studies in experimental animals show that tDCS is safe (Liebetanz et al., 2009), and a systematic review found that adverse effects are mild and transient (Brunoni et al., 2011a).

Another important characteristic of tDCS is that it can potentially be adapted for home-use, which would bring about an important advance to the therapeutic field of brain stimulation (Priori et al., 2009). From a methodological perspective, it has a reliable sham method as compared with, for instance, rTMS. Such characteristics (ease of use, low cost, portability, safe, potent effects) render tDCS a sound device for further clinical research, either as a substitutive therapy or a complementary treatment for other interventions (drug therapy, physical therapy, psychotherapy, and so forth) (Brunoni et al., 2011c), especially considering patients that are unable or unwilling to receive standard treatments.

Nonetheless, tDCS clinical trials are still in their infancy. One possible reason is that tDCS use requires basic knowledge on a neural basis of electrical current fields and neuroscience. In fact, an incorrect electrode montage or stimulation of the “wrong” area might generate non-specific or even negative effects (Datta et al., 2010; Mahmoudi et al., 2010; Mendonca et al., 2011). Therefore, it is more difficult to observe positive clinical effects by serendipity – also because tDCS has presently no standard clinical use, all effects can only be observed through research. Further, tDCS trials are methodologically complicated due to attrition, since the protocols demand daily stimulation for 1–4 weeks. A possible solution would be to use portable devices – specific tailored caps could be assembled in for targeting only the desired scalp areas. Furthermore, tDCS may be a device with little commercial interest compared to other medicines or even rTMS – in fact, by being *too* affordable and with a limited possibility of patenting, more robust business ventures are easily discouraged to develop tDCS commercially. Not surprisingly, at the present time tDCS research is mainly conducted in academic settings, usually with public grants. Nevertheless, this scenario could rapidly change depending on whether effective parameters of stimulation and findings are shown in clinical research. Finally, a simple reason to explain the current stage of development of tDCS is timing. Clinical trials, as well as the reporting and dissemination of results, usually has a significant time span.

Considering such challenges, we proposed a Research Topic in *Frontiers in Psychiatry*, named *The frontiers of clinical research on tDCS in neuropsychiatry*. The results were surprisingly positive, with 22 articles from new and experienced research groups that, considered together, represent a robust contribution to the advancement of the field. We are also grateful to all the reviewers – many of them productive researchers in the field – for their invaluable help in making suggestions that ultimately improved the manuscripts significantly. The articles hereby presented are divided in five main sections – in the first one, the neurobiological effects of tDCS are reviewed (Medeiros et al., 2012) and original articles on the electrophysiological effects of tDCS on visuo-spatial working memory (Heimrath et al., 2012), human color discrimination (Costa et al., 2012), and motor cortical excitability (Chaieb et al., 2012) are presented. The second section contains original articles exploring the behavioral effects of tDCS such as on the saccade task (Kanai et al., 2012), automatic verbal processes (Vannorsdall et al., 2012), working memory (Jones and Berryhill, 2012), emotional processing (Nitsche et al., 2012) and production of untruthful responses (Fecteau et al., 2012), and one review by Brasil-Neto (2012) on tDCS’ effects in learning and memory. The third section shows original articles on the clinical effects of tDCS on tinnitus (De Ridder and Vanneste, 2012), major depressive disorder (Blumberger et al., 2012; Knotkova et al., 2012) and pain (DosSantos et al., 2012), and reviews its effects on Alzheimer's disease (Hansen, 2012), stroke (Adeyemo et al., 2012; Madhavan and Shah, 2012), and smoking addiction (Fraser and Rosen, 2012). The fourth section presents computational theoretical models of tDCS for further application in clinical practice (Datta et al., 2012; Neuling et al., 2012; Sadleir et al., 2012). The last section reviews the application of spinal tDCS (Cogiamanian et al., 2012).

Moving tDCS research from bench to bedside has significant challenges. Nevertheless, there are opportunities for tDCS development as pharmacotherapy is reaching an efficacy and safety plateau and there are still unmet demands for the treatment of several disorders. tDCS therefore represents an interesting alternative that can offer additional therapeutic gains with a minimum of or no side effects. Whether the obstacles of clinical trials are solved or not, this collection of articles presented in this Research Topic provides promising evidence that tDCS could rise in the near future as a novel therapeutic tool and have a significant impact n psychiatry and neurorehabilitation.
